# Evaluation of Attachment Style and Social Support in Patients With Severe Migraine. Applications in Doctor-Patient Relationships and Treatment Adherence

**DOI:** 10.3389/fneur.2021.706639

**Published:** 2021-07-12

**Authors:** Rose-Angélique Belot, Margaux Bouteloup, Magalie Bonnet, Anne-Laure Parmentier, Eloi Magnin, Frédéric Mauny, Fabrice Vuillier

**Affiliations:** ^1^Laboratoire de Psychologie, Université Bourgogne Franche-Comté, Besançon, France; ^2^INSERM CIC1431 Centre d'Investigation Clinique Besançon, Besancon, France; ^3^Département de Neurologie, Centre Hospitalier Universitaire de Besançon, Besançon, France; ^4^UMR6249 Chrono Environnement, Besancon, France; ^5^Laboratoire d'Anatomie, Université de Franche-Comté, Besancon, France

**Keywords:** headaches and migraines, attachment, care, psychological factors, social support

## Abstract

**Objectives:** The aim of this observational study was to describe social support and patterns of attachment among patients with migraine. We hypothesized that in comparison to the general population, insecure attachment is overrepresented in migraine patients, and that these patients have less social support. We also aimed to study the specific relationship between attachment and social support. We hypothesized that patients with an insecure attachment style have less social support than patients with a secure attachment style.

**Methods:** A total of 101 consecutive patients (88.1% women) aged between 25 and 60 (average age = 41.4) were recruited at the Specialized Center for the Consultation of Primary Headaches at the Regional University Hospital Center of Besançon (France). Migraine impact and disability were evaluated using the Headache Impact Test (HIT-6) questionnaire and Migraine Disability Assessment (MIDAS) questionnaire. Patients also completed several self-administered psychological questionnaires in their validated French versions: the Medical Outcome Survey 36-Item Short-Form Health Survey, the Cungi Scale, the State-Trait Anxiety Inventory, the Beck Depression Inventory, the Relationship Scales Questionnaire and the Sarason's Social Support Questionnaire.

**Results:** The distribution of attachment profiles was different from that of the general population, with an overrepresentation of insecure attachment styles (*p* = 0.018). Our study showed that migraine patients had less social support than the general population, both in terms of the number of people providing support (*p* = 0.002) and the level of satisfaction concerning this social support (p = 0.000). We also found that neither the number of available persons score nor the satisfaction score were statistically different between the four attachment categories (*p* = 0.49). Patient's attachment style and social support influence the patient-doctor relationship, the therapeutic alliance and health behaviors such as treatment adherence.

**Conclusions:** Based on the data we obtained, we developed applications in patient care for people with particular attachment styles and low social support. A treatment plan adapted to the patient's attachment profile should be created to develop “precision medicine” using a personalized approach to the doctor-patient relationship. We would also recommend encouraging patients to participate in support groups, in order to strengthen their attachment systems and gain social support.

**Clinical Trial Registration:** https://clinicaltrials.gov/ct2/show/NCT03577548, identifier NCT03577548.

## Introduction

Migraine is a common disabling, recurrent headache disorder with a negative effect on quality of life. It affects nearly 15% of the global population between the ages of 22 and 55 years (1, 2). The multifactorial origin of migraine includes genetic, psychological and environmental factors, which leads to a complex pathophysiology. Psychological studies have reported the role of anxiety and depression (2, 3). Other psychological factors such as stress, coping, attachment and social support have also been studied, focusing on the level of disability (4–7).

The attachment styles that characterize human relationships have been described using two main theoretical models (8). The first model differentiates between three attachment styles: secure, preoccupied and avoidant (9). The second model subdivides the avoidant style into fearful and dismissing (10). In this four-style model, each style is defined by the relationship the subject has with themselves and others (10). In the general population, 50% of people have a secure attachment style, 24% dismissing, 15% fearful and 11% preoccupied (11). Adults with a secure attachment style feel confident with others and with themselves. In contrast, adults with insecure attachment styles are excessively worried about their relationships or do not care about having close relationships. Depending on the style of insecurity, they have a negative working model of themselves, others, or both. Subjects with a dismissing attachment style have a positive working model of themselves but are not confident that others will help them. Their insecurity extends to their surroundings and their environment. Conversely, subjects with a preoccupied attachment style have a negative working model of themselves, but a positive working model of others. They have an intense need to be listened to and understood, and they have a tendency to idealize others. Finally, people with a fearful attachment style have the most difficulty in relationships, as they have a negative working model of both themselves and others. They delay seeking help, do not trust others, and view themselves as undeserving.

Several studies have demonstrated that attachment style influences the level of disability in migraine patients (6, 12). Rossi et al. showed that insecure attachment is the most significant predictor of higher levels of disability in patients suffering from episodic migraine (13). In their statistical analysis, they found that attachment style influenced the MIDAS score by 20%. These studies used the three-category model and compared heterogeneous populations such as patients with migraine and patients with epilepsy (12) or different types of headaches (6, 13). However, none of these comparisons provided information that was precise enough to identify the psychological factors that lead to difficulties in treating these patients. Meredith et al. developed the Attachment Diathesis Model of Chronic Pain, which supports the association between attachment style and chronic pain, particularly related to experience of and adjustment to the pain (14). This model helps to prevent and reduce chronic pain in patients considered at risk, i.e., insecure patients.

It has been shown that migraine patients benefit from less social support than the general population (7, 15). Martin and Soon reported that migraine patients were significantly less satisfied with the social support available than control subjects were (15). Moreover, the migraine patient group gave significantly lower scores than the control group for the quality of the four dimensions of social support (appraisal, self-esteem, belonging and tangible).

It has also been reported in literature that attachment style and social support are linked. In the study by Khodarahimi et al. (16), perceived social support was positively or negatively correlated to the attachment depending on the style. For example, social support was positively associated with a secure attachment style and negatively correlated with a preoccupied attachment style.

Attachment and social support may affect the doctor-patient relationship (17, 18), patient adherence to treatment, success of therapeutic strategies (19) and symptom reporting (20). For example, there is evidence that patients with an insecure attachment style may present the same level of anxiety and depression before and after a multidisciplinary pain management program (21). This shows the need to know the patient's attachment style and social support in order to provide effective care.

The primary aim of our study was therefore to describe the attachment style and social support of patients with migraine who visited a tertiary hospital center, and compare these to the general population, to better understand the characteristics of this group of patients and consider whether this could be a factor in therapeutic failure. We hypothesized that in our sample, in comparison to the general population, insecure attachment would be overrepresented and migraine patients would have low social support. A secondary aim of our study was to study the specific relationship between attachment and social support. We hypothesized that patients with an insecure attachment style have less social support than patients with a secure attachment style.

We also evaluated anxiety, stress, depression and quality of life to ensure that our sample was representative of migraine patients consulting at hospital as described in literature (2–5). We therefore expected that our group of migraine patients would be more anxious, stressed and depressed, and have a lower quality of life than the general population.

## Materials and Methods

Our observational study, conducted between May 2016 and May 2018, was approved by the local clinical ethics board and the French Protection of Persons and Property Committee. All the necessary legal authorizations were obtained and the study was registered (NCT: 03577548). All patients gave their written informed consent.

### Participants

**Figure 1:**
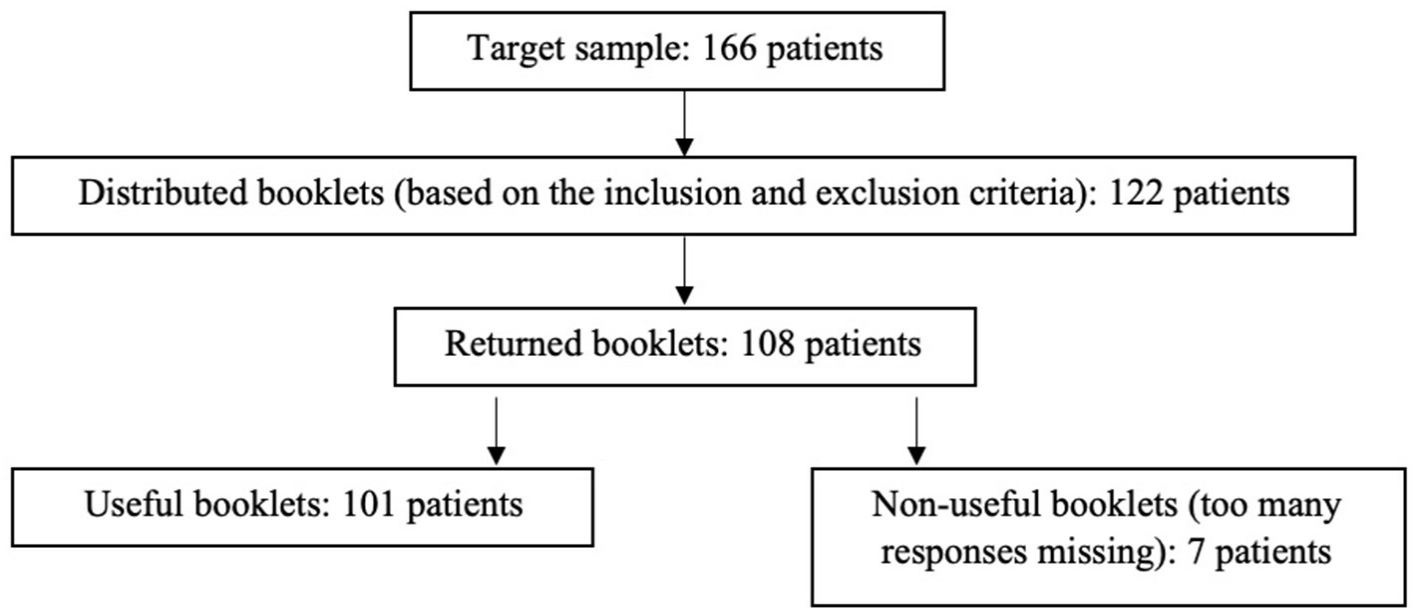
Flowchart.

A total of 101 consecutive patients (Flowchart, Figure 1), between 25 and 60 years old, were recruited at the Specialized Center for Primary Headaches in the Neurology Department of the Regional University Hospital Center of Besançon (France). We chose to include adult patients aged over 25 years because evidence in the literature suggests that the developmental period extends from age 12–25 (22).

We included patients with episodic migraine (EM) with or without aura, or chronic migraine (CM) with or without medication overuse, were included in the study. These conditions were based on the new International Classification of Headache Disorders criteria (previously ICHD-II, now ICHD-III beta version) (23). All patients who were pregnant at the time of the study or who had a history of psychiatric disorders were excluded from the study.

Socio-demographic data including sex, age, educational level and professional status was collected for all patients.

Instead of using a control group in our study, we compared our sample to the general population because all psychological variables explored in our study were assessed using norm-referenced tests, with norms obtained from the general population using large sample sizes of 200 to 6498 people (11, 24).

### Migraine Impact and Disability

We collected the following data from the 3 months prior to the study: average number of headache days per month, average duration of headache episodes, and average pain intensity. For pain intensity reporting, we used a visual analog 11-point scale, with 0 as “no pain” and 10 as “worst possible pain.” We also collected information concerning patients' preventive/background therapy and over-medication.

Migraine impact and disability were evaluated using the French versions of the Headache Impact Test – 6th version (HIT-6) questionnaire (25) and Migraine Disability Assessment (MIDAS) questionnaire (26). Patients were assigned one of four impact grades based on the HIT-6 score: grade 1: score ≤ 49; grade 2: score 50–55; grade 3: score 56–59; grade 4: score ≥ 60.

Based on the MIDAS score, patients were assigned one of four disability grades: score of 0–5: grade I, little or no disability; score of 6–10: grade II, mildly limiting disability; score of 11–20: grade III, moderately limiting disability; score > 20: grade IV, severely limiting disability.

### Psychological Procedure

The patients also completed a booklet of self-administered questionnaires.

The booklet consisted of the following questionnaires in their validated French versions: the Medical Outcome Survey 36-Item Short-Form Health Survey (MOS SF-36) (27), the Cungi Scale (24), the State-Trait Anxiety Inventory (STAI) (28), the Beck Depression Inventory (BDI) (29), the Relationship Scales Questionnaire (RSQ) (30) and Sarason's Social Support Questionnaire (SSQ6) (31).

The MOS SF-36 (27) is a standardized questionnaire used to assess patient health across eight dimensions. Four dimensions measure general physical health (physical functioning, role limitations due to physical health, pain, general health) and four other dimensions measure general mental health (role limitations due to emotional problems, energy/fatigue, social functioning, emotional well-being). Two main scores are available to summarize these scales: Physical Composite Score and Mental Composite Score. Each of the eight dimensions are scored on a 0–100 scale, while composite scores are norm based. Higher scores reflect better quality of life.

The Cungi Scale (24) is a French questionnaire used to measure how a person perceives stressors and stress. Subjects were asked to indicate their level of agreement or disagreement on a 6-point scale from 1 (not at all) to 6 (extremely). Stress is then classified into four levels: very low stress (score 12–19), low stress (score 20–30), high stress (score 31–45), and very high stress (score 45–72). In France, the average score is 24.59.

The State-Trait Anxiety Inventory (STAI) (28) is a questionnaire that assesses anxiety level based on state anxiety (A-state) and trait anxiety (A-trait). State anxiety measures how the person feels at a precise moment: it refers to a temporary condition when confronted with specific situations. Trait anxiety refers to a personality characteristic. The STAI uses a 4-point Likert scale (1 = almost never to 4 = almost always). A higher score indicates higher anxiety. In France, the norm-based score is 50.

The shortened Beck Depression Inventory (29) measures characteristic attitudes and symptoms of depression. Subjects choose the statement best describing how they felt over the past week for each of 13 items. Statements are scored from 0 (indicating little distress) to 3 (indicating much distress). Depression is evaluated in four levels: no depression (score 0–4), mild depression (score 5–7), moderate depression (score 8–15) and severe depression (score 15 or greater). In France, 0.4% of the population suffer from mild depression, 4.2% suffer from moderate depression and 3.2% suffer from severe depression.

**Figure 2:**
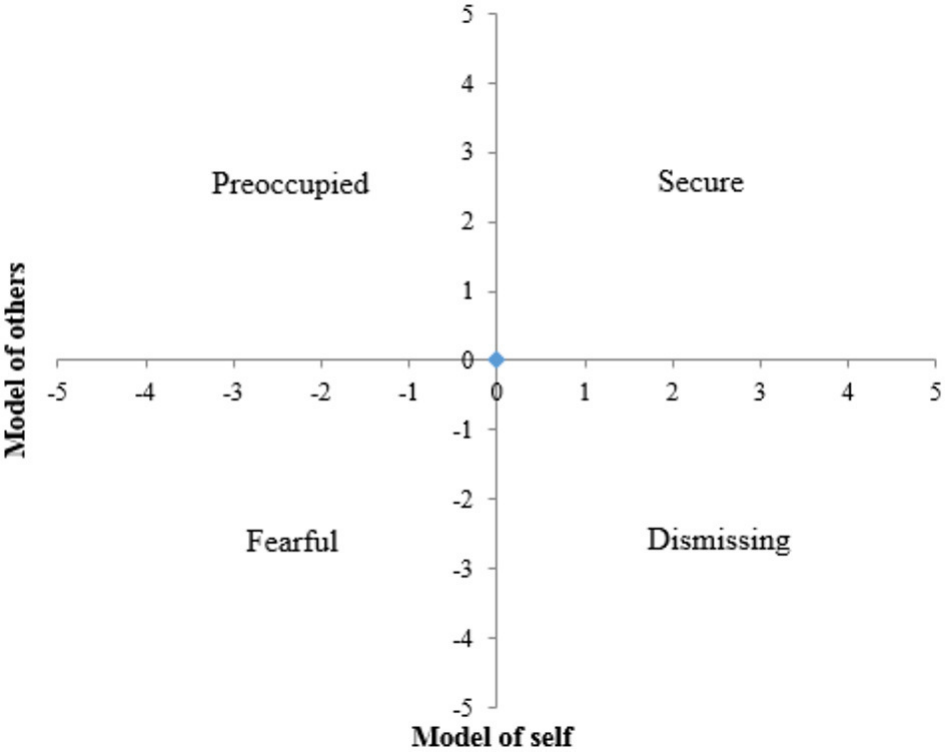
Model of the four attachment styles.

The Relationship Scales Questionnaire (RSQ) (30) was developed by Griffin and Bartholomew and measures attachment with two dimensions: model of self and model of others. Each dimension can be positive or negative (Figure 2), giving four categories of attachment can be defined: secure (positive model of both self and others); dismissing (positive model of self and negative model of others); preoccupied (negative model of self and positive model of others); fearful (negative model of both self and others).

The Sarason's Social Support Questionnaire (31) (SSQ6) is a questionnaire designed to measure the subject's perceptions of social support and level of satisfaction. Two scores are calculated based on six questions: the availability score (number of people) and the satisfaction score (from 1 = extremely dissatisfied to 6 = very satisfied). The total availability score is the sum of the number of people cited for each question. The total satisfaction score is the sum of the satisfaction score given for each question. In France, the average total availability score is 20.6 and the average total satisfaction score is 29.4 (31).

### Statistical Analyses

All analyses were performed using R version 3.5.0. All statistical tests were two-tailed and a *p*-value < 0.05 was considered statistically significant.

Quantitative variables are presented as mean ± standard deviation (SD) and qualitative variables as number and percentage. The Chi-squared or the Fishers exact tests was used to compare categorical variable and the Student *t*-test was used for quantitative variables. The comparison of the means between our sample and the data of the general population is carried out using a one-sample *t*-test. The comparison of the distribution of the numbers between our sample and the data of the general population are carried out using a Chi^2^ test or an exact Fisher test. Additionally, one-way analysis of variance (ANOVA) was used to examine differences in social support between the participants in the four categories of attachment style.

## Results

The socio-demographic and migraine characteristics for the final sample of 101 patients are reported in Tables 1, 2 respectively. In our cohort, women were overrepresented (sex ratio = 7.13) The average age of participants was 41.4 years. All marital statuses and levels of study were represented. The demographic data of our migraine patient group concerning age and sex is representative of a francophone migraine population (32) and the distribution of educational levels and family statuses in our sample is comparable to that of the general population (33).

**Table 1:** Descriptive statistics of sociodemographic data.

	**Patients (*N* = 101)**
**Sex**	
Female	88.1%
Male	11.9%
**Age** (years): mean **±** SD	41.4 **±** 9.8
**Educational level**	
Primary school	1%
NVQ	12.9%
BTEC	9.9%
A-levels	21.8%
BTEC HND	18.8%
Bachelor's degree	13.9%
Higher than Bachelor's degree	21.8%
**Family status**	
Single	9.9%
Unmarried couple	28.7%
Married	47.5%
*PACS* (Civil union)	13.9%

*NVQ, National Vocational Qualification; BTEC, Business and Technology Education Council; PACS, A registered civil union in France*.

**Table 2:** Descriptive statistics of headache characteristics.

	**Patients**	**Episodic migraine**	**Chronic migraine**	**Comparison EM/CM**	***p*-value**
	**(*N* = 101)**	**(*N* = 62)**	**(*N* = 39)**		
**Type of migraine**					
MwoA	64.4%	61.3%	69.2%	χ2 = 5.038	0.087
MwA	20.8%	27.4%	10.3%		
MwA/MwoA	14.9%	11.3%	20.5%		
**Medication overuse**	25.7%	0%	66.7%	χ2 = 52.229	**0.000**
**Already used a preventive therapy**	50.3%	46.8%	56.4%	χ2 = 0.889	0.35
**Intensity of pain with analog visual scale (0–10)**	7.5 **±** 1.3	7.5 **±** 1.2	7.5 **±** 1.4	T = −0.084	0.93
**MIDAS**: mean **±** SD	28.8 **±** 42.7	19.5 **±** 23.4	43.7 **±** 59.5	T = 2.431	**0.019**
Grade I	27.7%	32.3%	20.5%	χ2 = 8.76	**0.033**^a^
Grade II	11.9%	17.74%	2.6%		
Grade III	14.9%	12.9%	18.0%		
Grade IV	45.5%	37.1%	59.0%		
**HIT-6**: mean **±** SD	60.6 **±** 8.2	60.5 **±** 8.5	60.9 **±** 7.8	T = 0.249	0.80
Grade 1	9.9%	9.7%	10.3%	χ2 = 3.825	0.29^a^
Grade 2	10.9%	14.5%	5.1%		
Grade 3	11.9%	8.1%	18.0%		
Grade 4	67.3%	67.7%	66.7%		

*MwoA: migraine without aura/MwA: migraine with aura*.

a *Test exact de Fisher. Bold values mean the result is statistically significant*.

Analysis of the headache characteristics showed that all the migraine patients experienced severe symptoms according to the HIT-6 and MIDAS mean scores. EM patients presented severe symptoms in terms of frequency, duration or intensity of headache episodes. There was therefore no significant difference between EM and CM groups in terms of migraine impact and quality of life. The mean score for the HIT-6 was also similar in the two groups (HIT-6_EM = 60.5; HIT-6_CM = 60.9; *p* = 0.80). Moreover, there was no significant difference in the distribution of the HIT-6 grades (*p* = 0.29). However, for the MIDAS, there was a significant difference in the distribution of the grades (*p* = 0.033) and the mean scores (MIDAS_EM = 19.5; MIDAS_CM = 43.7; *p* = 0.019). This as we would expect for a group of EM patients compared to a group of CM patients, and shows that the patients had been correctly identified as belonging to the CM or EM group. These results overall show that quality of life is altered to the same extent for EM patients as for CM patients. This is consistent with a population of migraine patients who present with a disabling illness that leads them to seek a consultation at a regional expert referral center.

Psychological characteristics and quality of life compared to standards are reported in Table 3. Patients were stressed (Cungi Scale = 35.7; *p* = 0.000) and anxious (STAI_State = 54.4; *p* = 0.001), and more than half had symptoms of depression (*p* = 0.000). These results confirm that our sample corresponded to the standard description of a migraine population (2–5).

**Table 3:** Psychological characteristics and quality of life: comparisons with standards.

	**Average score – study patients**	**Standard**	**Statistical test result**	***p*-value**
**Quality of life (SF-36)**	(*N* = 101)	(*N* = 3,617)		
Physical functioning	89.6 **±** 15.0	84.5 **±** 21.2	T = 3.4	**0.008**
Role limitation, physical	53.5 **±** 39.7	81.2 **±** 32.2	T = −6.9	**0.000**
Pain	55.8 **±** 23.1	73.4 **±** 23.7	T = −7.7	**0.000**
General health	58.5 **±** 15.7	69.1 **±** 18.6	T = −6.8	**0.000**
Energy/fatigue	47.3 **±** 19.9	59.9 **±** 18.1	T = −6.4	**0.000**
Social functioning	62.5 **±** 21.4	81.6 **±** 21.4	T = −8.9	**0.000**
Role limitation, emotional	69.0 **±** 37.5	82.1 **±** 32.2	T = −3.5	**0.001**
Emotional well-being	57.7 **±** 17.8	68.5 **±** 17.6	T = −6.1	**0.000**
**Distress (Cungi)**	(*N* = 96)	(*N* = 206)		
Score: mean **±** SD	35.7 **±** 9.5	24.8 **±** 6.7	T = 11.3	**0.000**
**Anxiety (STAI)**	(*N* = 100)	(*N* = 200)		
State score: mean **±** SD	54.4 **±** 12.7	50 **±** 10	T = 3.5	**0.001**
Trait score: mean **±** SD	50.1 **±** 11.8	50 **±** 10	T = 0.4	0.97
**Depression (BDI)**	(*N* = 96)	(*N* = 16,883)	χ2 = 917.4	**0.000**^**a**^
Mild depression	20.8%	0.4%		
Moderate depression	27.1%	4.2%		
Severe depression	5.2%	3.2%		

a *Test exact de Fisher. Bold values mean the result is statistically significant*.

**Figure 3:**
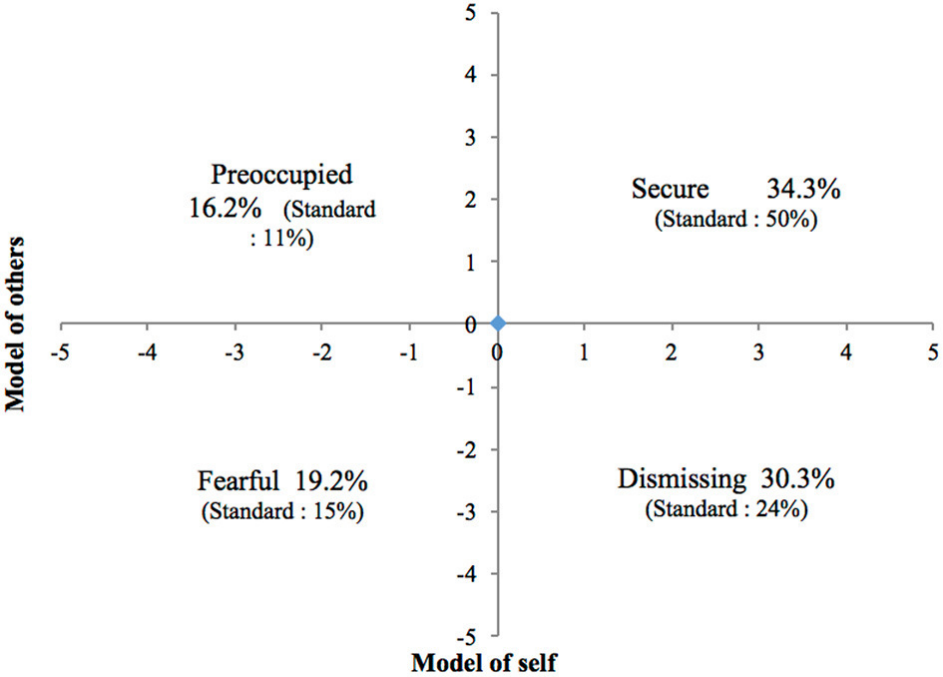
Distribution of attachment styles in our study.

Migraine patients benefited had less social support than the general population (SSQ6): the availability and satisfaction scores were below the norm (e.g., SSQ6-N = 17.95, *p* < 0.001; SSQ6-S = 26.55, *p* < 0.001). Additionally, the distribution of attachment styles in our sample was significantly different from that of the general population (*p* = 0.018) (Table 4 and Figure 3). Secure attachment was significantly underrepresented (50% vs. 34.3% in the general population), and the three sub-categories of insecure attachment were homogeneously overrepresented compared to standards (migraine patients: preoccupied = 16.2%; fearful = 19.2%; dismissing = 30.3% / general population: preoccupied = 11%; fearful = 15%; dismissing = 24%).

**Table 4:** Attachment style and social support: comparisons with standards.

	**Patients**	**Standards**	**Statistics**	***p*-value**
**Social support (SSQ6)**	(*N* = 98)	(*N* = 869)		
Number score: mean **±** SD	17.9 **±** 8.2	20.6 **±** 11.4	T = −3.2	**0.002**
Satisfaction score: mean **±** SD	26.5 **±** 7.5	29.4 **±** 4.8	T = −3.7	**0.000**
**Attachment (RSQ)**	(*N* = 99)	(*N* = 4454)	χ2 = 10.05	**0.018**
Secure	34.3%	50%		
Preoccupied	16.2%	11%		
Fearful	19.2%	15%		
Dismissing	30.3%	24%		

*Bold values mean the result is statistically significant*.

There was no significant difference in the average SSQ6 scores (number of subjects available and satisfaction) between the different attachment styles (Tables 5, 6). However, the SSQ6 scores revealed a noticeable difference between our cohort of migraine patients and the general population: for our study population, the number of people in the support network ranged from 17–19 (vs. an average of 20.6 in the general population in France), and the satisfaction score ranged from 25–28 (vs. an average of 29.4 in the general population in France).

**Table 5:** Mean SSQ6 (Number Score) of participants categorized into each of the four attachment styles.

**Attachment style**	**SSQ6 – *N* score (Mean)**	***F***	***p*-value**
Secure (34.3%)	17.03	0.356	0.79
Preoccupied (16.2%)	18.67		
Fearful (19.2%)	17.72		
Dismissing (30.3%)	19.13		

**Table 6:** Mean SSQ6 (Satisfaction Score) of participants categorized into each of the four attachment styles.

**Attachment style**	**SSQ6 – *S* score (Mean)**	***F***	***P*-value**
Secure (34.3%)	26.72	0.813	0.49
Preoccupied (16.2%)	26.84		
Fearful (19.2%)	28.61		
Dismissing (30.3%)	25.23		

## Discussion

The results of our study confirm the hypothesis that, in comparison to the general population, insecure attachment is overrepresented in patients with severe migraine and these patients have less social support. Only 34.3% of migraine patients have a secure attachment style, compared to 50% in the general population. Additionally, patients with severe migraine have less social support in terms of the number of people available (SSQ6-N = 17.9 *p* = 0.002) and satisfaction with the received social support (SSQ6-S = 25.6, *p* = 0.000).

Our results invalidate our second hypothesis (that the social support will be different depending on attachment style), as neither the number of social supports nor the satisfaction score were statistically different between the four attachment categories (SSQ6-N: *p* = 0.79; SSQ6-S: *p* = 0.49).

Patients with a secure attachment style are more able to benefit from medical treatment, to develop a therapeutic alliance with the doctor, and to ask for help and support from others (20, 34). The other 65.7% of our patients presented an insecure attachment style, with a distribution pattern between the three insecure attachment styles that corresponded to that of the general population. This distribution is consistent with a cohort of subjects consulting at a referral center.

In our study, patients with a dismissing-insecure attachment were the most common (30.3%), however these represent only 24% of the general population. During consultations, these patients may minimize their pain and appear to be restricted emotionally. They often exhibit exasperation or frustration and see others, such as doctors, as unhelpful. Patients with a dismissing attachment style experience more difficulties in medical follow-up and the therapeutic alliance (19, 20, 34).

The second subgroup of insecure attachment styles describes patients with a fearful attachment style; 19.2% of our cohort vs. 15% of the general population. They put off seeking help and tend to exacerbate the effects of the initial pain. They feel desperate, which could cause frustration for the doctor during follow-up because the patient takes a stance of helplessness and hopelessness (19, 20, 34).

The third subgroup are patients described as having a preoccupied attachment style. They represent 16.2% of our cohort, vs. 11% of the general population. In this category, the doctor-patient relationship can be positive, and patients may be invested and committed to their treatment, but their lack of confidence in their own capacity to adapt and change can damage the therapeutic alliance and treatment adherence (19, 20, 34).

Given that nearly 70% of migraine patients consulting at the hospital present an insecure attachment style, a precise description of health behaviors according to attachment style gives us a better understanding of the doctor/patient relationship, reasons for some difficulties in treating patients, and even therapeutic failures. The therapeutic alliance and process of building trust, which are necessary for the success of long-term treatment, will be affected by the patient's insecurity, whether the patient has a preoccupied, detached or fearful insecure attachment style.

Our study found that compared to the general population, migraine patients receive less social support in terms of quantity and are less satisfied with the support available. The study conducted by Blomkvist (7) concerning social support for migraine patients focused on comparing the social network and activities between migraine patients and cluster headache patients. They found that migraine patients benefitted more social support and closer social contacts than cluster headache patients. Our study is therefore innovative, because we compared migraine patients to the general population and we were able to study two dimensions of social support; the number of people perceived as available for support, and satisfaction with the social support.

Nevertheless, it would also be interesting to investigate the nature of the social support received by migraine patients in more detail and identify the areas in which they are unsatisfied. Social support has a different impact depending on whether it is provided by a health professional or a close friend/relative. In the context of close personal relationships, emotional support is expected, whereas it seems that a more information-based support is more effective in the doctor-patient relationship (35).

Study of social support according to the four attachment styles reveals that there is no significant difference in terms of quality: patients with a secure attachment style do not have a better quality of social support than patients with dismissing, preoccupied or fearful attachment styles (Tables 5, 6). This is surprising because a previous study reported that perception of social support may depend on attachment style (16). We had therefore initially expected patients with a secure attachment style to be more satisfied with their perceived social support. However, our results may be related to the small sample size of each category of attachment style. This result merits investigation on a wider scale.

Finally, insecure attachment and less social support could explain some difficulties encountered by migraine patients in care, treatment adherence, and follow-up. Indeed, the literature has shown that attachment style affects the way in which the patient experiences the doctor/patient relationship, their confidence in the medical profession, and their ability to adhere to a long-term treatment plan (19). Identifying the available social support is therefore necessary in order to tailor the care pathway (hospitalization vs. outpatient care).

It could also provide insight into how the patient experiences the initial consultation, the importance of which was pointed out in the FRAMIG-3 and GRIM studies (36, 37). For instance, the FRAMIG-3-study concluded that satisfaction with the first consultation is a pivotal factor in migraine patients' decision whether or not to continue consultation (36). The dialogue with the practitioner also appeared to be a key element of care. According to attachment theory, a patient's satisfaction with the first consultation and the expectations about the relationship with the practitioner differ depending on whether or not the patient presents a secure, dismissing, fearful or preoccupied attachment style (14, 19).

To our knowledge, our study is the first to evaluate attachment style in migraine patients using the four categories defined using the RSQ. We hope to use this categorization to better understand the expectations the patient has of their consultation, the factors that influence the doctor/patient relationship, treatment adherence and the patient's way of reporting symptoms (16–20).

It can be difficult for doctors to treat patients who have an insecure attachment style: either they engage in their therapy hesitantly due to a lack of trust (dismissing attachment style), or they engage ambivalently, which leads to failure of a therapy that was originally asked for (fearful and preoccupied styles). Integrating these patients into support groups alongside their treatment could prove to be particularly beneficial as this could increase both self-confidence and confidence in others, and would provide adequate support. Support groups would allow patients to modify and strengthen their attachment systems, gain social support, and maintain a better adherence to their treatment (38). Having the opportunity to meet with other patients who have had similar experiences, and also with expert patients, could help these patients to improve their self-confidence and their working models of themselves and others.

## Conclusion

Our study seemed to show that patients with severe migraine consulting in a tertiary headache center have a specific distribution of attachment profiles that is different from the general population, with an overrepresentation of insecure attachment styles. Compared to the general population, our study showed that migraine patients have less social support, both in terms of the number of people providing support and the level of satisfaction concerning this social support.

These factors should be taken into account when developing a care plan for these patients, because they entail different health behaviors and expectations about the care provided, and they influence the doctor-patient relationship. Moreover, these results may have significant implications in clinical practice, especially concerning the therapeutic alliance and patient adherence to treatment.

A care plan tailored to each patient's attachment profile should be created to develop “precision medicine” that uses a personalized approach to the patient-doctor relationship, especially during the first consultation (36). We would also recommend a different treatment approach, in which patients also participate in support groups with other migraine patients, in order to strengthen their working models of themselves and others and benefit from more social support. The implementation of these recommendations may lead to improved treatment adherence, an increase in patients' follow-up care and a reduction in treatment failures.

## Limitations

Our study has some limitations. Firstly, we did not use a control group because we decided compare our results to standards. Secondly, we recruited patients in a tertiary headache center. Our results therefore do not allow us to understand the psychological factors involved among patients with less severe forms of migraine because in our center we primarily encountered those with severe forms of episodic migraine.

## Data Availability Statement

The raw data supporting the conclusions of this article will be made available by the authors, without undue reservation.

## Ethics Statement

The studies involving human participants were reviewed and approved by Clinical Trial Registration Number: NCT03577548. The patients/participants provided their written informed consent to participate in this study.

## Author Contributions

R-AB participated in planning the research project, data collection, data analysis, and writing of the article. MBou participated in the data collection, data analysis, and writing of the article. MBon participated in setting up the research project, data collection, data analysis, and writing of the article. A-LP and EM participated in the data analysis and writing of the article. FM participated in setting up the project and in the writing of the article. FV supervised the study and participated in setting up the project, data collection, data analysis, and the writing of the article. All authors contributed to the article and approved the submitted version.

## Conflict of Interest

The authors declare that the research was conducted in the absence of any commercial or financial relationships that could be construed as a potential conflict of interest.
